# Publication bias in animal research: a systematic review protocol

**DOI:** 10.1186/2046-4053-2-23

**Published:** 2013-04-27

**Authors:** Matthias Briel, Katharina F Müller, Joerg J Meerpohl, Erik von Elm, Britta Lang, Edith Motschall, Viktoria Gloy, Francois Lamontagne, Guido Schwarzer, Dirk Bassler

**Affiliations:** 1 Basel Institute for Clinical Epidemiology and Biostatistics, University Hospital Basel, Basel, Switzerland; 2 Department for Clinical Epidemiology and Biostatistics, McMaster University, Hamilton, Canada; 3 Center for Pediatric Clinical Studies, University Children’s Hospital Tuebingen, Tuebingen, Germany; 4 Department of Neonatology, University Children’s Hospital Tuebingen, Calwer-Str. 7, Tuebingen, 72076, Germany; 5 German Cochrane Centre, Institute of Medical Biometry and Medical Informatics, University Medical Center Freiburg, Freiburg, Germany; 6 Cochrane Switzerland, IUMSP, University Hospital Lausanne, Lausanne, Switzerland; 7 Institute of Medical Biometry and Medical Informatics, University Medical Center Freiburg, Freiburg, Germany; 8 Clinical Research Centre Ètienne Le Bel and Department of Medicine, University of Sherbrooke, Sherbrooke, Canada

**Keywords:** Publication bias, Animal experimentation, Systematic review, meta-analysis, The OPEN Project

## Abstract

**Background:**

Systematic reviews and meta-analyses of pre-clinical studies, *in vivo* animal experiments in particular, can influence clinical care. Publication bias is one of the major threats of validity in systematic reviews and meta-analyses. Previous empirical studies suggested that systematic reviews and meta-analyses have become more prevalent until 2010 and found evidence for compromised methodological rigor with a trend towards improvement. We aim to comprehensively summarize and update the evidence base on systematic reviews and meta-analyses of animal studies, their methodological quality and assessment of publication bias in particular.

**Methods/Design:**

The objectives of this systematic review are as follows:

•To investigate the epidemiology of published systematic reviews of animal studies until present.

•To examine methodological features of systematic reviews and meta-analyses of animal studies with special attention to the assessment of publication bias.

•To investigate the influence of systematic reviews of animal studies on clinical research by examining citations of the systematic reviews by clinical studies.

Eligible studies for this systematic review constitute systematic reviews and meta-analyses that summarize *in vivo* animal experiments with the purpose of reviewing animal evidence to inform human health. We will exclude genome-wide association studies and animal experiments with the main purpose to learn more about fundamental biology, physical functioning or behavior.

In addition to the inclusion of systematic reviews and meta-analyses identified by other empirical studies, we will systematically search Ovid Medline, Embase, ToxNet, and ScienceDirect from 2009 to January 2013 for further eligible studies without language restrictions.

Two reviewers working independently will assess titles, abstracts, and full texts for eligibility and extract relevant data from included studies. Data reporting will involve a descriptive summary of meta-analyses and systematic reviews.

**Discussion:**

Results are expected to be publicly available later in 2013 and may form the basis for recommendations to improve the quality of systematic reviews and meta-analyses of animal studies and their use with respect to clinical care.

## Background

Pre-clinical research has its main purpose in enhancing our understanding of physiologic and pathologic processes. However, many pre-clinical studies, *in vivo* animal experiments in particular, may also influence clinical care of patients through the following mechanisms: i) information for the design of clinical studies, ii) clinical guidelines considering pre-clinical evidence when clinical evidence is lacking, or iii) direct guidance of clinical practice. For instance, in the widely accepted Surviving Sepsis Guidelines recommendations regarding target arterial blood pressure for vasopressor use and positive end-expiratory pressure in the prevention of lung injury, draw on results from animal experiments [1]. In addition, a recent empirical study showed that a substantial number of overviews of animal experiments investigating therapies for sepsis explicitly extrapolated results from animal studies to human patients and that subsequent citations of these overviews in publications of clinical studies reflect the influence of pre-clinical on clinical research [2].

Whenever a summary of the current evidence is needed for decision-making in clinical research, health-care policy, or clinical practice, systematic reviews (SRs) and, where appropriate, meta-analyses are most useful tools. SRs offer a structured and transparent way to comprehensively identify and evaluate the available evidence on a specific topic. Meta-analyses increase the precision and generalizability of effect estimates by quantitatively summarizing the results of individual studies included in an SR in order to provide a single best estimate with maximal statistical power [3]. SRs and meta-analyses of pre-clinical studies are still relatively rare in the medical literature. Mignini and Khan identified 30 SRs of laboratory animal experiments in 2006, and Peters *et al*. found 86 using a more sensitive search strategy and a broader definition of laboratory animal experiments [4,5]. However, in an update study Korevaar *et al*. reported that SRs of pre-clinical studies are getting more common over time, doubling their number roughly every 3 years between 1997 and 2010 [6].

The validity and usefulness of the results of SRs and meta-analyses strongly depend on their methodological rigor. Assessing bias, such as publication bias, is essential, when conducting SRs. If the publication or non-publication of research findings depends on the nature and direction of the results (definition of publication bias [7]) then the published studies are no longer a random sample of all studies that have been conducted, but constitute a biased sample leading to spurious summary results. Different methods have been developed to graphically and statistically investigate publication bias [8-12]. Although all these methods have their limitations one should still try to address the likelihood of publication bias in each SR as thorough as possible [9,13].

While principles of critically appraising SRs of clinical research are well established, their application to SRs of pre-clinical studies appears variable [2]. Previous empirical studies on SRs of animal experiments found that only 16% (5/30) and 37% (17/46) of meta-analyses considered publication bias [4,5]. Korevaar *et al*. reported that between 2005 and 2010 the proportion of meta-analyses of *in vivo* animal studies that assessed publication bias increased to 60% (21/35) [6]. A recently published survey conducted in animal laboratories in the Netherlands reported that just about 50% of animal experiments are published; employees of for-profit organizations even estimated that only 10% of animal experiments appear in peer-reviewed journals [14]. Lack of statistical significance was identified as one of several important reasons for non-publication.

Korevaar *et al*. conducted their search for SRs of animal experiments in 2009/10 [6]. It remains unclear how the number and methodological quality, in particular assessment of publication bias, of such SRs evolved up to the present (January 2013), and to what extent they are cited by clinical studies. We will therefore conduct a comprehensive SR summarizing the results from Peters *et al*. and Korevaar *et al*. and updating the evidence base for SRs of animal experiments. This SR will be part of the project, To Overcome failure to Publish nEgative fiNdings (OPEN Project), which was developed with the goal of elucidating the scope of publication bias and non-publication of studies through a series of SRs and policy evaluations (http://www.open-project.eu).

### Objectives

The specific goals of the present SR of animal studies are: determining the number of published SRs of animal studies up to the present; investigating methodological features of SRs and meta-analyses of animal studies especially with respect to assessment of publication bias, and investigating the influence of SRs of animal studies on clinical research by examining citations of the SRs in clinical studies.

## Methods

### Eligibility criteria

To allow for a valid combination of results we will use similar criteria and search strategies to Peters *et al*. and Korevaar *et al*. [5,6], to identify relevant SRs and meta-analyses of animal experiments. We will consider an article an SR if it provides details on the source(s) of evidence searched and information on at least one of the following: i) search terms used, ii) any limitation placed on the search, or iii) inclusion and exclusion criteria. In addition, we will use a second, more stringent, definition of an SR requiring besides a systematic search and explicit inclusion and exclusion criteria, also a focused research question and a systematic evaluation of the quality of the included studies [15]. For meta-analyses, a review has to report on some form of quantitative synthesis of results of more than one animal experiment. SRs and meta-analyses will be included if they summarize *in vivo* animal experiments with the purpose of reviewing animal evidence to inform human health and if they can be allocated to one of the following categories: i) investigation of the efficacy of a medical or surgical intervention; ii) investigation of the side-effects or toxicity of a medical intervention; iii) investigation of the mechanisms of action of a medical intervention; iv) investigation of risk factors (epidemiological associations or mechanisms of action of disease); v) investigation of effects of an exposure to a chemical substance; vi) overview of animal models for disease, and vii) investigation of diagnostic test accuracy. Articles including human studies in addition to animal studies will be considered too. As did Korevaar *et al*. we will exclude genome-wide association studies and animal experiments with the main purpose to learn more about fundamental biology, physical functioning or behavior and not to inform human healthcare.

### Search strategy

We will systematically search electronic databases such as Ovid Medline, Embase, Toxnet (http://toxnet.nlm.nih.gov/; including Toxline, DART, and HSDB) and ScienceDirect, all from 2009 to the present (January 2013). In addition, the bibliographies of any eligible articles identified will be checked for additional references. No language restrictions will be applied. The search strategy used by Korevaar *et al*. has been slightly modified with the support of a librarian/information specialist. The full search strategies are displayed in Additional file 1. We will not search any grey literature (that is, literature that has not been formally published).

### Study selection

Two reviewers will independently and in duplicate screen titles and abstracts of search results. If a title and abstract cannot be rejected with certainty by both reviewers, the full text of the paper will be retrieved and assessed for eligibility. Any disagreements among reviewers will be resolved by discussion and consensus or, if needed, third party arbitration.

### Data extraction

A specifically designed data extraction form will be developed and pilot-tested. Working in teams of two, we will independently extract relevant information from each eligible article. From all eligible SRs (those included in Peters *et al*. or Korevaar *et al*. plus those identified through our search update) we will abstract the following: first author and publication year; journal (goal: distinguish clinical from other journals); whether the article identifies itself as an SR or meta-analysis in the title or abstract; the objective; search strategy (how many and which electronic databases, language restrictions, if any grey literature was searched, if yes, which and how); whether any formal assessment of study quality was performed, and which instrument was used; number of included studies; whether any funding sources were reported for the SR and abstracted from the included studies; whether any assessment of the presence of publication bias was reported, and which method was used; any other comments on publication bias; whether any reporting guideline (QUOROM, PRISMA) for SRs was mentioned.

From meta-analyses we will additionally extract the following data: number of included studies and total number of experiments; number and species of animals used; whether intention to treat was mentioned; whether effect estimates from individual studies were reported; which method was used for data synthesis; whether heterogeneity was assessed.

Any disagreements will be resolved by discussion and consensus or, if needed, third party arbitration. We will use the ISI Web of Science Internet-based citation database to identify clinical publications citing included animal SRs. We will not do this for all included SRs but will rather focus on the following sample: SRs with meta-analysis published between 2005 and 2009 (29 articles) and a random sample of 29 SRs without meta-analysis. Limiting to articles published until 2009 allows all articles to be available in the literature for at least 3 years until our analysis. We will focus on studies likely to influence clinical decision making, such as i) randomized controlled trials; ii) matched controlled trials; iii) crossover trials; iv) uncontrolled prospective trials; v) guidelines; vi) retrospective cohort studies; vii) prospective cohort studies; viii) laboratory experiments with healthy human volunteers; ix) cross-sectional surveys, and x) case report/case series. Two reviewers will review each of the 58 studies independently and in duplicate. They will abstract the role of the citation by allocating a citation to one of the following categories: i) use of citation unrelated to animal studies in review; ii) used citation to provide at least partial justification for the study or a future study; iii) used citation to support or explain their findings; iv) used citation to discuss physiological pathways, and v) used citation to justify the measure of certain variables.

### Data reporting

Data reporting will involve a descriptive summary of meta-analyses and systematic reviews. We will report the study according to PRISMA guidelines [16].

## Discussion

This SR aims to comprehensively summarize the epidemiology and methodological quality of SRs and meta-analyses of *in vivo* animal studies with special attention to the assessment of publication bias. SRs of animal studies can influence clinical care directly or indirectly through guidelines and clinical studies that are designed based on evidence from pre-clinical studies. In order to better understand the extent of that influence we plan to examine citations of identified reviews in publications of clinical studies.

Our protocol has strengths and limitations. The strengths are that we will use a comprehensive approach to identify SRs and meta-analyses of *in vivo* animal studies through a sensitive search strategy and inclusion of previously identified articles. We will employ two definitions of SRs: a broader one that ensures comparability with previous empirical studies and a more stringent definition that will be applied to all articles meeting the broader criteria. A limitation of our protocol is that our results might be affected by publication bias because we will not search any grey literature. We are therefore limiting the generalizability of our results about methodological quality and publication bias to the published SRs of *in vivo* animal studies.

This SR together with the results of other SRs in the context of the OPEN Project will raise awareness about the widespread plague of publication bias and the complexity of this issue. Moreover, these reviews will serve as a foundation for a recommendations workshop, which will enable key members of the biomedical research community (for example, funders, research ethics committees, journal editors, et cetera.) to develop future policies and guidelines to reduce the frequency of non-publication and related biases.

## Abbreviations

OPEN: to Overcome Failure to Publish Negative Findings; PRISMA: Preferred Reporting Items for Systematic reviews and Meta-Analyses; SR: systematic review.

## Competing interests

We declare that all authors and contributing members have no competing interests.

## Authors’ contributions

MB, JM, and DB conceived of the study. MB, VG and EM designed the search strategies. MB drafted the manuscript with the help of KM, JM and DB. EvE, BL, EM, VG and GS critically reviewed the manuscript for important intellectual content. All authors read and approved the final version before submission. MB, JM and DB are guarantors.

## Supplementary Material

Additional file 1 Search strategy. Click here for file
