# Supplementary material for: Publication bias in animal research: a systematic review protocol
Source: Syst Rev. 2013 Apr 27;2:23. doi: 10.1186/2046-4053-2-23 (PMC3651300; doi:10.1186/2046-4053-2-23)
Supplement: Additional file 1 — Search strategy. [file 2046-4053-2-23-S1.docx]

Appendix 1

Ovid MEDLINE(R) search strategy (2009 to 2013)

| **#** | **Searches** | **Comments** |
| --- | --- | --- |
| 1 | meta-analysis.pt. | Meta-analysis, systematic review |
| 2 | (meta-analy* or metaanaly* or metanaly* or met analy*).ti,ab. |  |
| 3 | "meta-analysis as topic"/ |  |
| 4 | 1 or 2 or 3 |  |
| 5 | review.pt. |  |
| 6 | review.ab. |  |
| 7 | 5 or 6 |  |
| 8 | systematic.ti,ab. |  |
| 9 | 7 and 8 |  |
| 10 | (systematic* adj5 (review* or overview*)).ti,ab. |  |
| 11 | 4 or 9 or 10 |  |
| 12 | (comment or editorial or letter).pt. | Excluding comment,editorial, letter |
| 13 | 11 not 12 |  |
| 14 | ("2009" or 201*).yr. | Restrict to Publication year 2009 to current |
| 15 | 13 and 14 |  |
| 16 | limit 15 to animals | Includes articles indexed with animals or both animals AND humans |
| 17 | exp Animal Experimentation/ | Animal experiments |
| 18 | exp models, animal/ |  |
| 19 | experiment*.ti,ab. |  |
| 20 | limit 19 to animals |  |
| 21 | 17 or 18 or 20 |  |
| 22 | 15 and 21 | Syst Rev, Meta-anal and animal experiment |
| 23 | 16 or 22 | Final result |

ScienceDirect search strategy (2009 to 2013)

Search Fields: Title, Abstract, Keywords

| **Queries: Advanced Search Mode** | **Results (ScienceDirect translation of advanced search query)** |
| --- | --- |
| **Systematic W/6 review and animal*** *(W/6: the two words in any order within a maximum of 6 words)* | **117** articles found for: pub-date > 2008 and TITLE-ABSTR-KEY(**systematic W/6 review**) and TITLE-ABSTR-KEY(**animal***) |
| **Metaanaly* and animal*** | **3** articles found for: pub-date > 2008 and TITLE-ABSTR-KEY(**metaanaly***) and TITLE-ABSTR-KEY(**animal*)** |
| **“Meta-analy*” and animal*** *(****“Meta-analy*”*** *or* ***“Meta analy*”*** *finds identical results. " - " is ignored.)* | **137** articles found for: pub-date > 2008 and TITLE-ABSTR-KEY(**"meta-analy*"**) and TITLE-ABSTR-KEY(**animal*)** |

Embase search strategy (2009 to 2013)

| #43 | #36 AND #41 AND [2009-2013]/py |
| --- | --- |
| #42 | #36 AND #41 |
| #41 | #37 OR #38 OR #39 OR #40 |
| #40 | 'systematic review'/exp |
| #39 | systematic*:ab,ti AND review*:ab,ti |
| #38 | 'meta analysis'/exp |
| #37 | meta AND analy*:ab,ti |
| #36 | #21 AND #35 |
| #35 | #22 OR #23 OR #24 OR #25 OR #26 OR #27 OR #28 OR #29 OR #30 OR #31 OR #32 OR #33 OR #34 |
| #34 | 'side effect':lnk |
| #33 | 'adverse drug reaction':lnk |
| #32 | 'drug toxicity':lnk |
| #31 | 'carcinogen'/exp |
| #30 | carcinogen*:ab,ti |
| #29 | 'mutagenic agent'/exp |
| #28 | mutagen*:ab,ti |
| #27 | 'comparative toxicology'/exp |
| #26 | 'genetic toxicology'/exp |
| #25 | 'toxicology'/exp |
| #24 | toxicology:ab,ti |
| #23 | 'environmental exposure'/exp |
| #22 | 'environmental exposure':ab,ti |
| #21 | 'animal experiment'/exp OR (animal:ab,ti AND stud*:ab,ti) OR 'animal model'/exp OR (animal:ab,ti AND model*:ab,ti) |

ToxNet search strategy (2009 to 2013) including Toxline, DART, and HSDB

| 1 | Systematic Review AND Animal |
| --- | --- |
| 2 | Meta-analysis AND Animal |
